# Neonatal infection leads to increased susceptibility to Aβ oligomer-induced brain inflammation, synapse loss and cognitive impairment in mice

**DOI:** 10.1038/s41419-019-1529-x

**Published:** 2019-04-11

**Authors:** Paula S. Frost, Fernanda Barros-Aragão, Rachel T. da Silva, Aline Venancio, Isadora Matias, Natalia M. Lyra e Silva, Grasielle C. Kincheski, Pedro M. Pimentel-Coelho, Fernanda G. De Felice, Flávia C. A. Gomes, Sergio T. Ferreira, Claudia P. Figueiredo, Julia R. Clarke

**Affiliations:** 1 School of Pharmacy, Federal University of Rio de Janeiro, Rio de Janeiro, RJ 21944-590 Brazil; 2 Institute of Biomedical Sciences, Federal University of Rio de Janeiro, Rio de Janeiro, RJ 21944-590 Brazil; 3 Federal University of Santa Catarina, Florianópolis, SC 88040-900 Brazil; 4 Institute of Medical Biochemistry Leopoldo de Meis, Federal University of Rio de Janeiro, Rio de Janeiro, RJ 21944-590 Brazil; 5 Institute of Biophysics Carlos Chagas Filho, Federal University of Rio de Janeiro, Rio de Janeiro, RJ 21944-590 Brazil; 6 Department of Psychiatry, Centre for Neuroscience Studies, Queen’s University, Kingston, ON Canada

## Abstract

Harmful environmental stimuli during critical stages of development can profoundly affect behavior and susceptibility to diseases. Alzheimer disease (AD) is the most frequent neurodegenerative disease, and evidence suggest that inflammatory conditions act cumulatively, contributing to disease onset. Here we investigated whether infection early in life can contribute to synapse damage and cognitive impairment induced by amyloid-β oligomers (AβOs), neurotoxins found in AD brains. To this end, wild-type mice were subjected to neonatal (post-natal day 4) infection by *Escherichia coli* (1 × 10^4^ CFU/g), the main cause of infection in low-birth-weight premature infants in the US. *E. coli* infection caused a transient inflammatory response in the mouse brain starting shortly after infection. Although infected mice performed normally in behavioral tasks in adulthood, they showed increased susceptibility to synapse damage and memory impairment induced by low doses of AβOs (1 pmol; intracerebroventricular) in the novel object recognition paradigm. Using in vitro and in vivo approaches, we show that microglial cells from *E. coli*-infected mice undergo exacerbated activation when exposed to low doses of AβOs. In addition, treatment of infected pups with minocycline, an antibiotic that inhibits microglial pro-inflammatory polarization, normalized microglial response to AβOs and restored normal susceptibility of mice to oligomer-induced cognitive impairment. Interestingly, mice infected with by *E. coli* (1 × 10^4^ CFU/g) during adolescence (post-natal day 21) or adulthood (post-natal day 60) showed normal cognitive performance even in the presence of AβOs (1 pmol), suggesting that only infections at critical stages of development may lead to increased susceptibility to amyloid-β-induced toxicity. Altogether, our findings suggest that neonatal infections can modulate microglial response to AβOs into adulthood, thus contributing to amyloid-β-induced synapse damage and cognitive impairment.

## Introduction

Several experiences during the perinatal period, including stress, nutrition, trauma or infections may profoundly and persistently impact the developing brain and the associated behavioral outcomes^1–4^. Epidemiological and experimental data have shown that early exposure to different common pathogens is related to an increased incidence of autism-spectrum disorders^5^ and schizophrenia^6^. However, to date little is known about the influence of early life infections in the development of inflammatory neurodegenerative diseases in the elderly. In humans, a causal relation between neurodegenerative diseases and neonatal infections has proven difficult to establish due to the long time period between infection and the emergence of clinical symptoms^7^.

Sporadic Alzheimer’s disease (AD) is the most common neurodegenerative condition worldwide^8^. Although aging remains the most important risk factor for AD, increasing evidence suggest that cumulative environmental factors and poor lifestyle habits in different life stages contribute to disease development^9–12^. Inflammation is a common denominator to several of these AD risk factors. Besides, it is now known that inflammation plays a central and dual role in Alzheimer’s pathology:^13^ while it is initially beneficial for clearance of extracellular aggregates of amyloid-β peptide (Aβ)^14^, central toxins that accumulate in AD brains^15^, the sustained pro-inflammatory profile can be extremely deleterious to neuronal function^16^.

Here, we investigate whether an early-life infection increases susceptibility to cognitive impairment induced by low doses of amyloid-β oligomers (AβOs), neurotoxins found in AD brains during initial stages of the disease^17^. Wild-type mice were subjected to neonatal (post-natal day 4) infection by *Escherichia coli*, the main cause of infection in low-birth-weight premature infants in the US^18^. Our results show that early life *E. coli* infection causes an inflammatory response in the mouse brain starting shortly after infection. Although infected mice perform normally in behavioral tasks in adulthood, animals show increased susceptibility to synapse damage and cognitive impairment induced by low doses of AβOs injected into the lateral ventricle. Using in vitro and in vivo approaches, we show that microglial cells from *E. coli*-infected mice undergo exacerbated activation when exposed to low doses of AβOs. In addition, blockage of neonatal infection-induced microglial polarization to a pro-inflammatory profile leads to normalization of behavioral and cellular responses to AβOs in adult mice. Finally, persistent priming of microglial cells is specifically associated to infections taking place during the neonatal period, since injection of *E. coli* at later stages of development (adolescence and adulthood) did not affect susceptibility of mice to AβO-induced cognitive impairment. Altogether, our findings suggest that neonatal infections can modulate microglial response to AβOs in adult mice, thus contributing to amyloid-β-induced synapse damage and cognitive impairment.

## Results

**Fig. 1:**
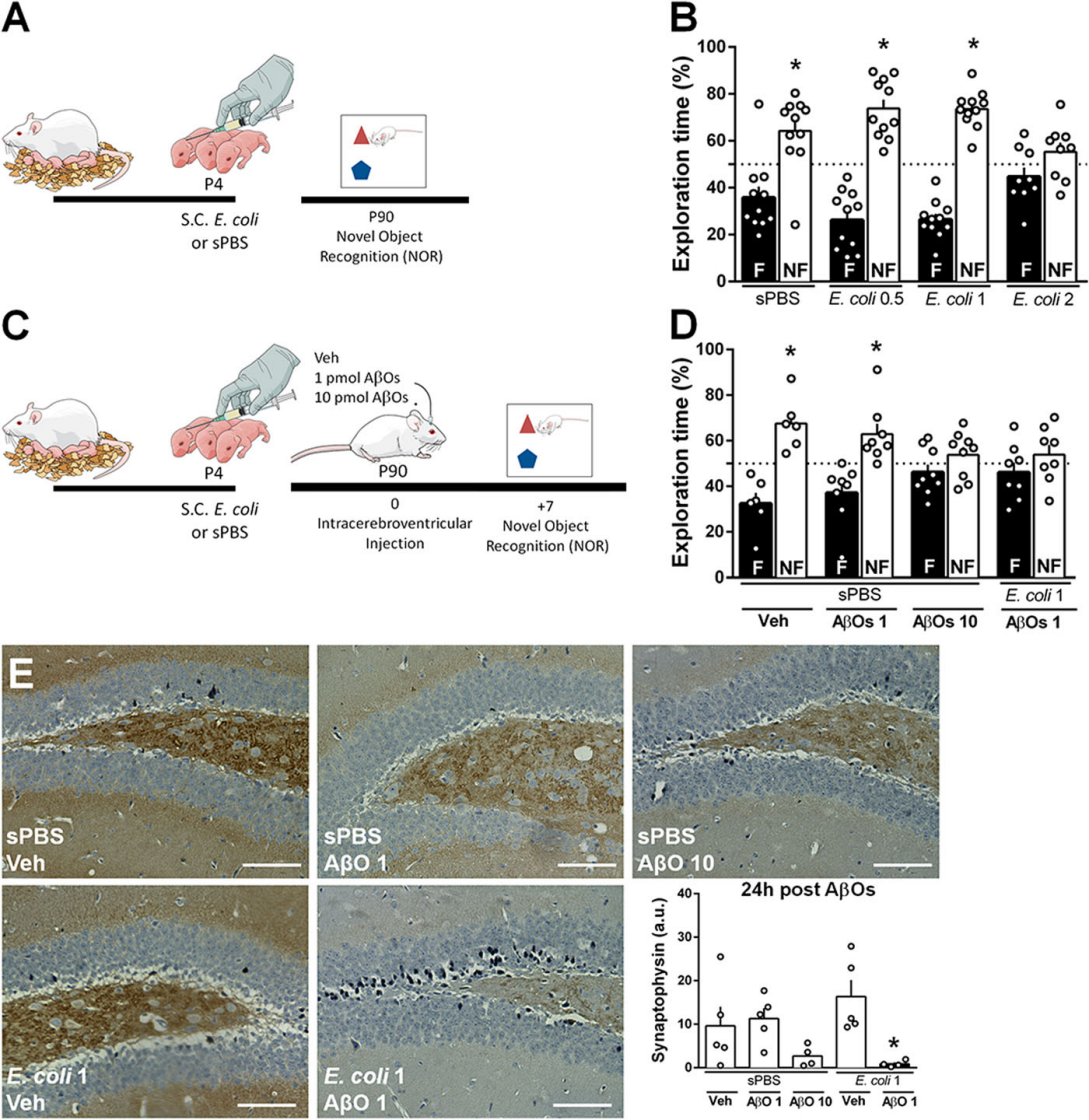
Neonatal *E. coli* infection increases susceptibility to AβO-induced cognitive impairment and synapse loss. **a** Swiss pups received a subcutaneous (s.c.) injection of sterile PBS (sPBS) or 0.5, 1 or 2 × 10^4^ CFU/g of body weight of *Escherichia coli* (*E. coli* 0.5, *E. coli* 1, *E. coli* 2, respectively) at post-natal day 4 (P4). At post-natal day 90 (P90), they were tested in the Novel Object Recognition (NOR) task (**b**). **c** Mice subjected to s.c. injection of 1 × 10^4^ CFU/g of *E. coli* or sPBS at P4, received an i.c.v. injection of vehicle (Veh), 1 pmol (AβOs 1) or 10 pmol of AβOs (AβOs 10) at P90, and were tested in NOR task (**d**) seven days later. Bars represent mean ± S.E.M. of percentage of time spent exploring the familiar (**f**; black bars) and non-familiar (NF; white bars) objects used in test session. **e** Representative images of synaptophysin immunoreactivity in the DG hippocampal region of mice subjected to *E. coli* 1 infection or sPBS at P4, and given an i.c.v. injection of vehicle, AβOs 1 or AβOs 10. Graph shows integrated immunoreactivity (optical density) for synaptophysin in the hippocampus. Scale bar: 50 µm. In **b**: *p = 0.0125 for sPBS and *p < 0.0001 for *E. coli* 0.5 and *E. coli* 1; in (**d**): *p = 0.0133 for sPBS + Veh and *p = 0.0258 for sPBS + AβOs 1, one-sample Student’s *t* test compared to fixed value 50. In (**e**): *p = 0.0396 Student’s t test compared to sPBS + AβOs 1

In order to investigate whether an early life inflammatory event modulates the susceptibility of the brain to AβO-induced toxicity, we developed a mouse model of early-life *E. coli* infection. Initially, we determined the amount of *E. coli* that induced no long-term memory deficits or mortality in Swiss mice. Therefore, male pups injected with 0.5; 1 or 2 × 10^4^ CFU/g of *E. coli* s.c. at post-natal day 4 (P4) were tested in the novel object recognition task (NOR) at 90 days of age (Fig. 1a). As expected, animals that received a s.c. injection of sterile PBS (sPBS) spent significantly more time exploring the non-familiar than the familiar object used in test session, indicating that they successfully learned the task (Fig. 1b). Conversely, male mice that received 2 × 10^4^ CFU/g of *E. coli* at P4 did not differentiate between familiar and novel objects presented in the test session (Fig. 1b, *E. coli* 2), indicating that this amount of bacteria induces persistent cognitive impairment *per se* in mice. Since lower amounts of *E. coli* (0.5 or 1 × 10^4^ CFU/g) had no persistent effect on cognition of adult mice (Fig. 1b; *E. coli* 0.5; *E. coli* 1), we chose the intermediate dose of *E. coli* (1 × 10^4^ CFU/g) for subsequent experiments. Curiously, female pups that received this amount of *E. coli* showed persistent cognitive impairment in the NOR task (Suppl. Fig. 1A–C) suggesting that early life infection may have sex-specific effects on the mouse developing brain.

We previously described that a single intracerebroventricular (i.c.v.) injection of 10 pmol of AβOs in mice results in impaired formation of NOR memory, whereas 1 pmol of AβOs i.c.v. have no effect on cognitive function^19^. Based on these previous findings, mice injected at P4 with *E. coli* 1 × 10^4^ CFU/g or sPBS received an i.c.v. injection of either 1 or 10 pmol of AβOs and were trained in the NOR task, at 90 days of age (Fig. 1c). In agreement to our previous studies^19,20^, control mice that received an i.c.v. injection of 10 pmol AβOs showed cognitive dysfunction (Fig. 1d). Interestingly, while 1 pmol of AβOs had no effect on cognitive performance of sPBS-treated mice, *E. coli* infected-mice infused with a single subtoxic dose of AβOs (1 pmol/site, i.c.v.) failed to differentiate between familiar and non-familiar objects, indicating an impaired NOR memory formation (Fig. 1d). The effects appear to be specific on memory formation, since no changes in locomotion (Suppl. Fig. 1D) or exploratory behavior (Suppl. Fig. 1E) were observed.

Synapse loss is a hallmark of AD and contributes to memory impairment in several conditions^21^. In order to investigate whether adult mice neonatally infected with *E. coli* are more susceptible to AβO-induced synapse loss, we quantified hippocampal levels of the pre-synaptic protein synaptophysin 24 h after i.c.v. injection of oligomers. We found that mice subjected to neonatal *E. coli* infection showed a marked decrease in hippocampal synaptophysin immunolabeling when treated with 1 pmol AβOs i.c.v., when assessed both 24 h (Fig. 1e) and 7 days after i.c.v. infusion of oligomers (Suppl. Fig. 2A–D), whereas in non-infected mice this subtoxic dose of amyloid-β had no effect on this synaptic protein. Altogether, these findings suggest that previous *E. coli* infection increases susceptibility to AβO-induced synapse damage and memory impairment.

Extensive evidence has shown that decreased maternal care during the neonatal period has persistent effects on adult behavior^22,23^. Therefore, we investigated whether *E. coli* infection at P4 induced changes in maternal behavior, which could explain changes in adult behavior following AβO injection. Litters injected with sPBS or *E. coli* 1 and their respective dams were daily observed to assess maternal behavior. No changes in the percentage of time spent by dams licking (Suppl. Fig. 3A) or feeding pups (Suppl. Fig. 3B) as well as the percentage of time spent at the nest (Suppl. Fig. 3C) were seen between groups. Moreover, no mortality (data not shown) or body weight loss (Suppl. Fig. 3D) were induced by this amount of *E. coli* compared to sPBS-treated mice. We then investigated whether s.c. injection of *E. coli* was associated to systemic infection in neonatal animals. Significant bacteremia were seen both 1 and 24 h after infection (Suppl. Fig. 4A, B).

**Fig. 2:**
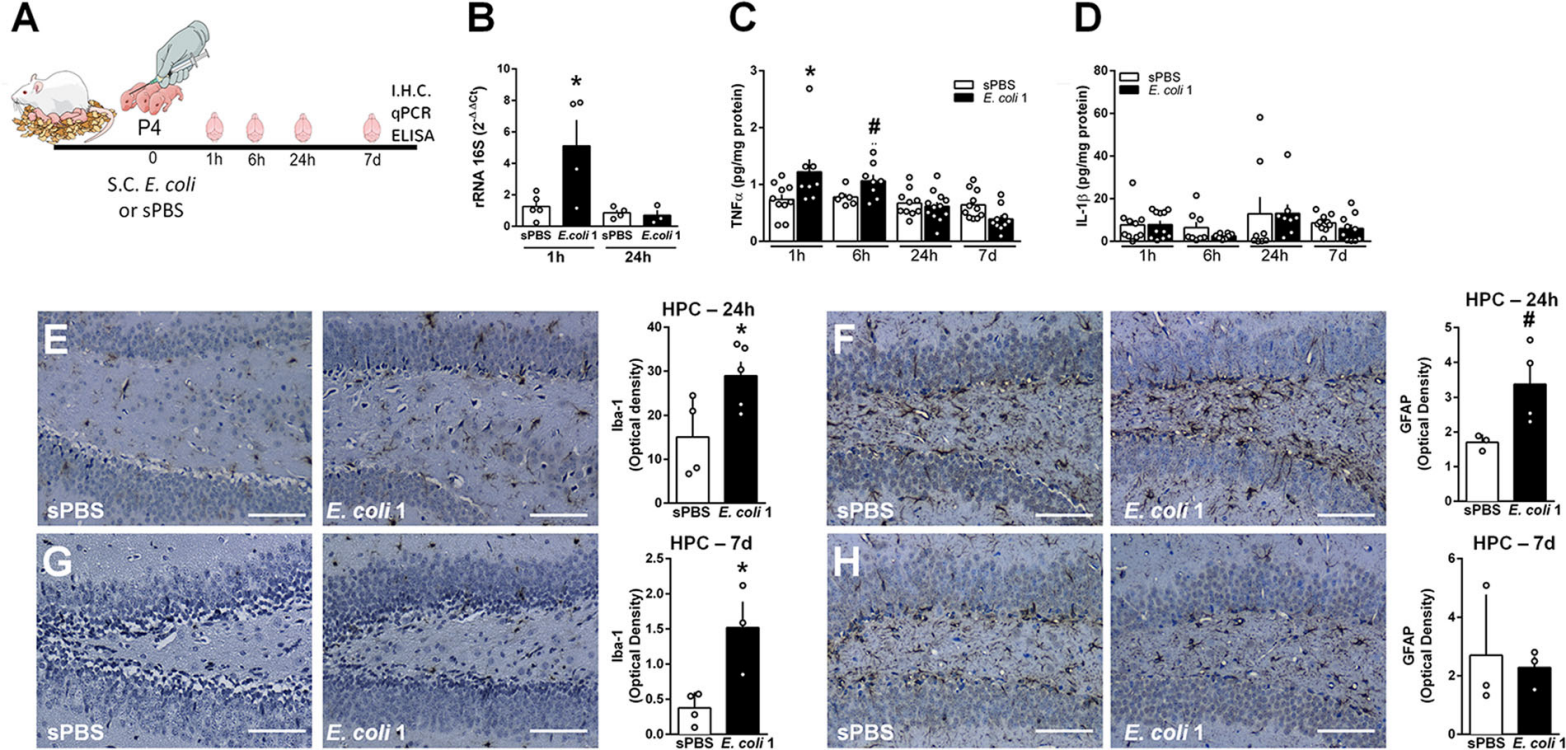
Neonatal *E. coli* infection induces transient hippocampal inflammation and astro- and microgliosis. **a** Swiss pups received a s.c. injection of sterile PBS (sPBS) or *E. coli* 1 × 10^4^ CFU/g (*E. coli* 1) at post-natal day 4 (P4), and brains were collected 1, 6, 24 h or 7 days later. **b** qPCR for gamma-proteobacteria rRNA 16 S was performed in whole brains of mice 1 or 24 h after s.c. injection of *E. coli* or sPBS, and normalized by actin. TNF-α (**c**) and IL-1β levels (**d**) were measured in brains of mice at different timepoints after s.c. injection of *E. coli* or sPBS. **e**, **g** Representative images of Iba-1 immunoreactivity in the DG hippocampal region of mice 24 h (**e**) and 7 days (**g**) after s.c. injection of *E. coli* 1 or sPBS. Graphs show integrated immunoreactivity (optical density) for Iba-1 in the hippocampus. **f**, **h** Representative images of GFAP immunoreactivity in the hippocampus of mice 24 h (**f**) and 7 days (**h**) after s.c. injection of *E. coli* 1 or sPBS. Graphs show integrated immunoreactivity (optical density) for GFAP in the hippocampus. Scale bars = 50 µm. In (**b**): *p = 0.037, in (**c**): *p = 0.0449, ^#^0.0576, in (**e**): *p = 0.037, in (**f**): ^#^*p* = 0.0575, in (**g**): *p = 0.0189, Student’s *t* test

Next, we investigated the effects of *E. coli* infection on the developing brain, in order to identify factors that could contribute to the increased susceptibility to AβO-induced toxicity seen in adulthood. To determine whether *E. coli* reaches the developing brain after s.c. injection, we performed qPCR in brain samples of mice after infection (Fig. 2a, b) for determination of gamma-proteobacteria 16 S ribosomal RNA (rRNA 16 S). High levels of bacterial RNA were found in brains of mice 1 h, but not 24 h, after infection at P4 (Fig. 2b). We hypothesized that the bacterial RNA detected in brain samples 1 h after infection corresponded to circulating *E. coli*. To address this issue, *E. coli*-infected neonatal mice were perfused with sterile saline before brain samples were collected and probed for rRNA 16 S by qPCR. Results were similar to those seen in non-perfused mice: increased levels of *E. coli* rRNA 16 S were found in brains of animals 1 h after infection (Suppl. Fig. 4C). We next investigated whether infection was leading to an increased permeability of the blood brain barrier, by measuring the presence of endogenous IgG in the brain parenchyma following *E. coli* infection^24^. No significant IgG staining was found 1 h after injection of *E. coli* when compared to brains of sPBS mice, whereas diffuse IgG staining was observed in only 1 brain out of three pups 24 h after infection (Suppl. Fig. 4D–H). Altogether, these findings suggest that s.c.-injected *E. coli* either enters the brain parenchyma without breaking the integrity of the blood brain barrier, or it binds to the epithelial lining of the choroid plexus and brain ventricles.

To further characterize the effects of neonatal *E. coli* infection, we evaluated the levels of pro-inflammatory cytokines in pups’ brain samples at different time intervals after neonatal sPBS or *E. coli* s.c. injections (Fig. 2a). In agreement, we further found that *E. coli* infection induced a transient increase (after 1 and 6 h) in brain levels of the pro-inflammatory cytokine tumor necrosis factor α (TNF-α) (Fig. 2c), whereas levels of interleukin 1β (IL-1β) were comparable between groups at the evaluated timepoints (Fig. 2d). We further found an increase in the immunoreactivity for the microglial marker Iba-1 (Fig. 2e) and a trend in increase of GFAP immunoreactivity (an astrocytic marker) (Fig. 2f) in the hippocampi of infected pups 24 h after bacterial infection when compared to sPBS-injected mice. In addition, while Iba-1 immunoreactivity remained higher in *E. coli*-infected pups after 7 days of infection (Fig. 2g), levels of GFAP (Fig. 2h) in infected pups were similar to control levels at this later timepoint, thus indicating that hippocampal astrocytes undergo a transient activation following *E. coli* infection.

**Fig. 3:**
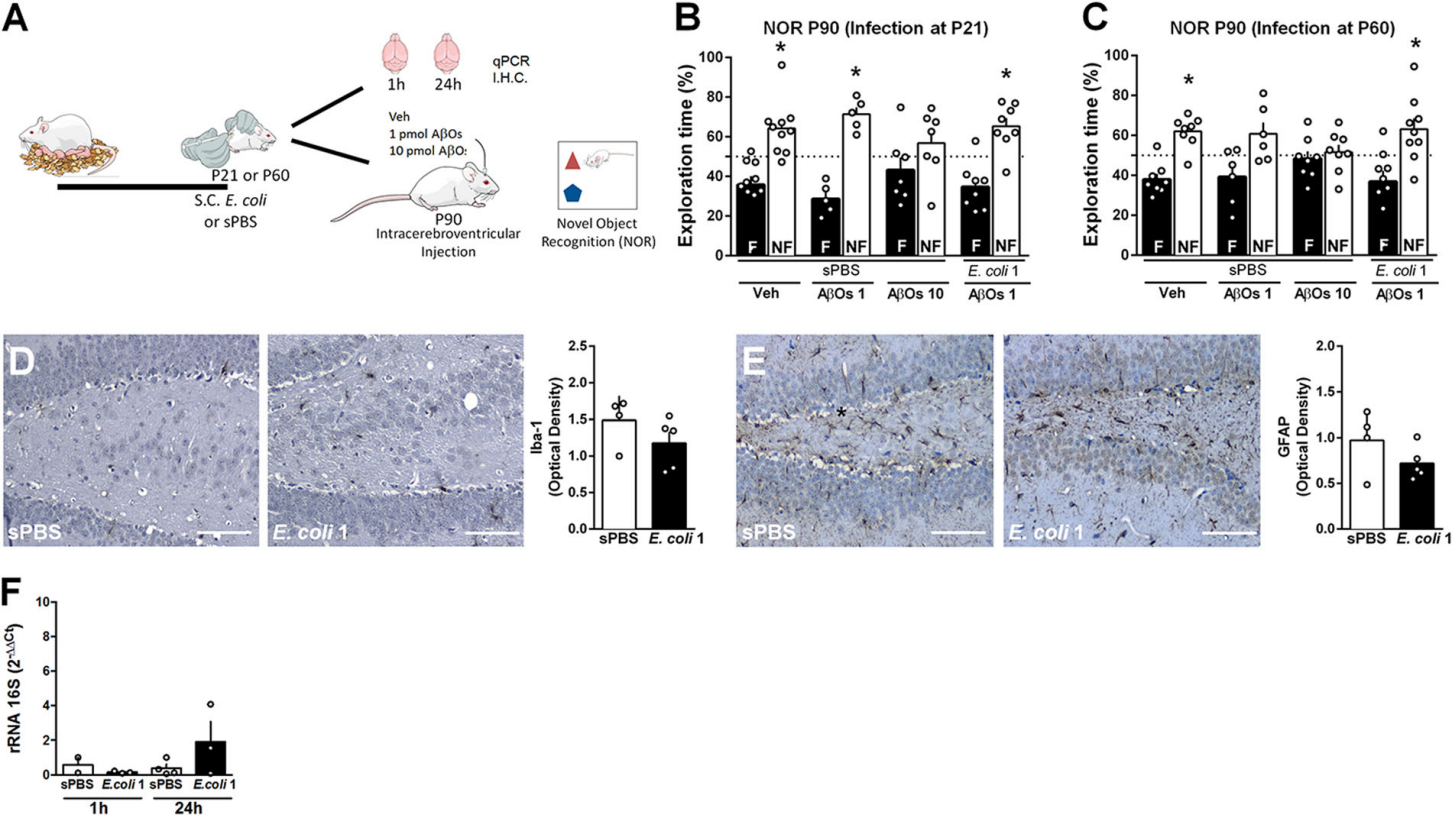
*E. coli* infection in adolescent or adult mice does not lead to increased susceptibility to AβO-induced cognitive impairment. **a** Mice injected s.c. with 1 × 10^4^ CFU/g of *E. coli* (*E. coli* 1) or sterile PBS (sPBS) at post-natal day 21 (P21) or at post-natal day 60 (P60), and received an i.c.v. injection of vehicle (Veh), 1 pmol (AβOs 1) or 10 pmol AβOs (AβOs 10) at P90. Seven days after i.c.v. injections, mice were tested in NOR task (**b**, **c**). Bars represent mean ± S.E.M. of percentage of time spent exploring the familiar (F; black bars) and non-familiar (NF; white bars) objects used in test session. **d** Representative images of Iba-1 immunoreactivity in the hippocampus of mice 24 h after injection of *E. coli* 1 or sPBS at P21. Graph shows integrated immunoreactivity (optical density) for Iba-1 in the hippocampus. Scale bar: 50 µm. **e** Representative images of GFAP immunoreactivity in the hippocampus of mice 24 h after injection of *E. coli* 1 or sPBS at P21. Graph shows integrated immunoreactivity (optical density) for GFAP in the hippocampus. Scale bar: 50 µm. **f** qPCR for gamma-proteobacteria rRNA 16 S was performed in whole brains of mice 1 or 24 h after s.c. injection of *E. coli* or sPBS in P21 mice, and normalized by actin. In (**b**): *p = 0.0091 for sPBS + Veh, *p = 0.0039 for sPBS + AβOs 1, *p = 0.009 for *E. coli* 1 + AβOs 1; in (**c**): *p = 0.0035 for sPBS + Veh, *p = 0.0423 for *E. coli* 1 + AβOs 1, Student’s *t* test compared to fixed value 50

Since neonatal *E. coli* infection resulted in an increased susceptibility to AβO-induced cognitive impairment in adulthood, we next asked whether this effect is restricted to infection at certain stages of neurodevelopment. To address this issue, we infected adolescent (P21) and adult mice (P60) with *E. coli* (s.c.; 1 × 10^4^ CFU/g), and at 90 days of age they received an i.c.v. injection of AβOs or vehicle (Fig. 3a). Interestingly, 1 pmol AβOs had no effect on NOR memory formation of animals infected with *E. coli* during these later stages of development (Figs. 3b, c; Suppl. Fig. 5A–D). Furthermore, *E. coli* infection at P21 had no effect on either Iba-1 (Fig. 3d) or GFAP (Fig. 3e) immunolabeling in the hippocampi of infected mice compared to control. To determine whether s.c.-injected *E. coli* reached the brain of adolescent mice, we performed qPCR for gamma proteobacteria rRNA 16 S subunit in brains of mice infected at post-natal day 21. In contrast to the brains of neonatal mice, no bacterial RNA was found either 1 or 24 h after s.c. infection in adolescent mice (Fig. 3f). These findings highlight that infections during the neonatal period have larger potential to cause long-lasting impact on brain and behavior.

**Fig. 4:**
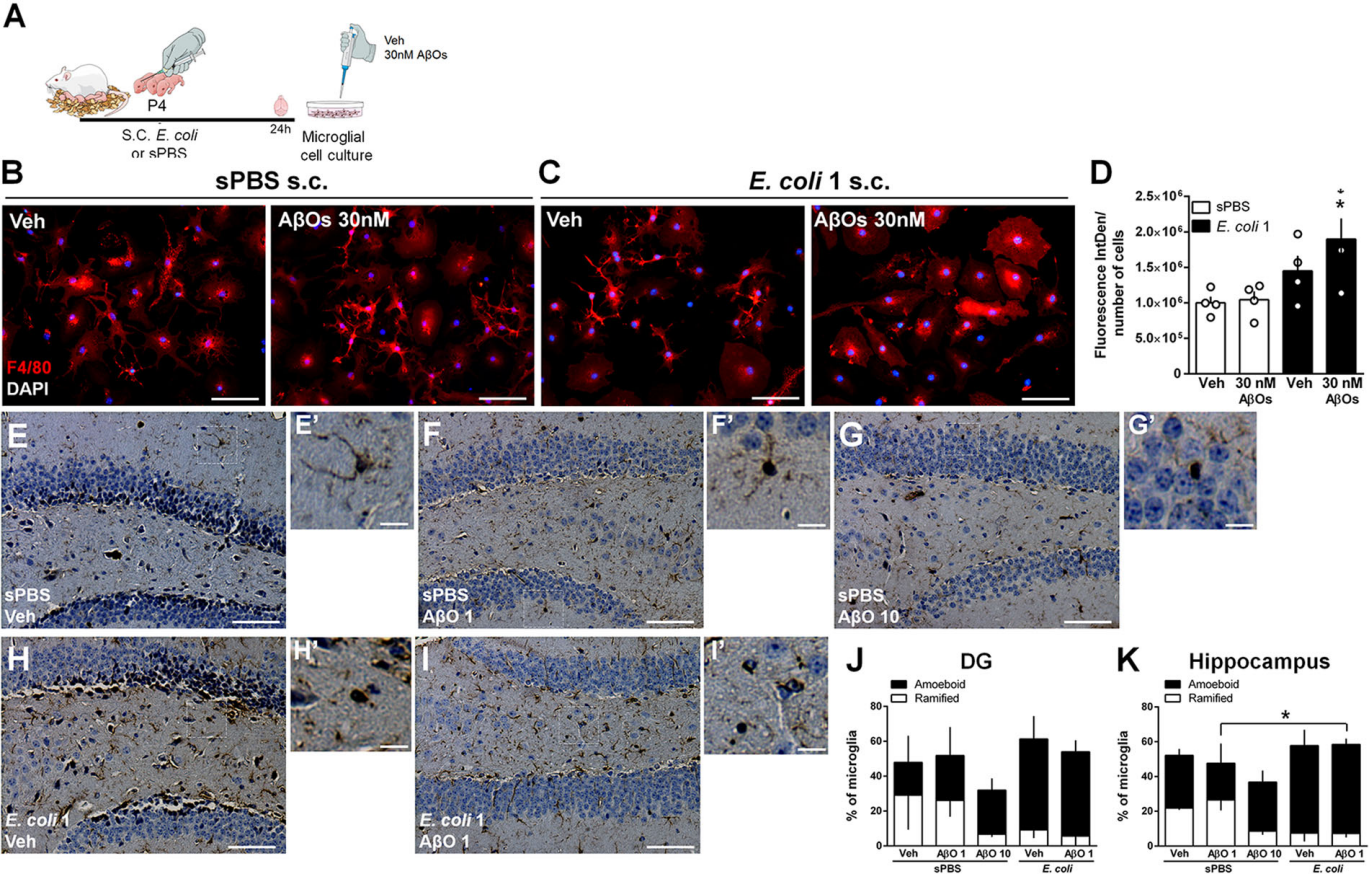
Microglial cells from neonatal *E. coli*-infected mice show increased susceptibility to AβOs. **a** Swiss pups received a s.c. injection of sterile PBS (sPBS) or *E. coli* 1 × 10^4^ CFU/g (*E. coli* 1) at post-natal day 4 (P4). Brains of mice were removed after 24 h and purified microglial cells were obtained and maintained in culture. After 14 days in culture, cells were treated with vehicle (Veh) or AβOs 30 nM for 24 h. **b**, **c** Representative images of immunocytochemistry performed using anti-F4/80 antibody in microglia derived from sPBS (**b**) and *E. coli*-injected pups (**c**). **d** Bars represent mean ± S.E.M. of fluorescence intensity normalized by the number of cells per field. Data are representative of four independent cultures (2 coverslips per experimental condition, 10 images per coverslip in each experiment). Scale bar: 60 µm. **e**–**I'** Representative images of Iba-1 immunoreactivity in the DG hippocampal subregion of mice subjected to *E. coli* 1 or sPBS s.c. injection at P4, and given Veh, 1 (AβO 1) or 10 pmol (AβO 10) of AβOs i.c.v. at P90. Graphs show the percentage of microglial cells that show an amoeboid or ramified morphology in the DG (**j**) or total hippocampus (**k**). Scale bar in (**e**, **f**, **g**, **h** and **i**): 50 µm; scale bar in (**e**’, **f**’, **g**’, **h**’ and **i**’): 20 µm. In (**d**): *p = 0.0405 one-way ANOVA followed by Tukey; in (**k**): *p = 0.0352 Student’s *t* test comparing indicated groups

Microglial priming is a phenomenon that involves an exaggerated or heightened microglial response to a second inflammatory stimulus^25^, and which has been linked to neurodevelopmental and neurodegenerative diseases^26^. We then investigated whether microglial priming could contribute to the exacerbated response of neonatally-infected mice to subtoxic amounts of AβOs. Microglia from *E. coli*-infected pups (*E. coli* 1 × 10^4^ UFC/g at P4) were isolated and maintained in culture (Fig. 4a). We found that microglial cells isolated from sPBS-infected mice at P4 showed a resting morphology in vitro (Fig. 4b). Treatment of these cultures with a subtoxic dose of AβOs (30 nM) did not induce either a significant alteration in cell morphology (Fig. 4b) or increased immunoreactivity of the microglia/macrophage marker F4/80 (Fig. 4b, d). Interestingly, microglial cells isolated from brains of *E. coli*-infected mice showed an increase in F4/80 immunoreactivity after exposure to 30 nM of AβOs (Fig. 4c, d). We next investigated whether microglial priming was also induced by neonatal infection in vivo, by performing a comparative investigation of microglial morphology in different hippocampal subregions of adult mice after i.c.v. injection of vehicle or of the subtoxic dose of AβOs (1 pmol). Among mice neonatally treated with sPBS, 1 pmol AβOs had no effect on the percentage of amoeboid and ramified microglial cells compared to Vehicle-injected mice (Figs. 4e–k, Suppl. Fig. 6). In contrast, the same dose of AβOs shifted microglial morphology into a predominantly amoeboid state in all hippocampal regions of *E. coli* infected mice (Figs. 4e–k, Suppl. Fig. 6). We also found that brains of infected mice showed predominance of amoeboid microglial cells even in the absence of AβOs (*E. coli*-vehicle injected mice), suggesting that infection *per se* induces persistent microglial morphological changes in vivo (Figs. 4e–k, Suppl. Fig. 6).

**Fig. 5:**
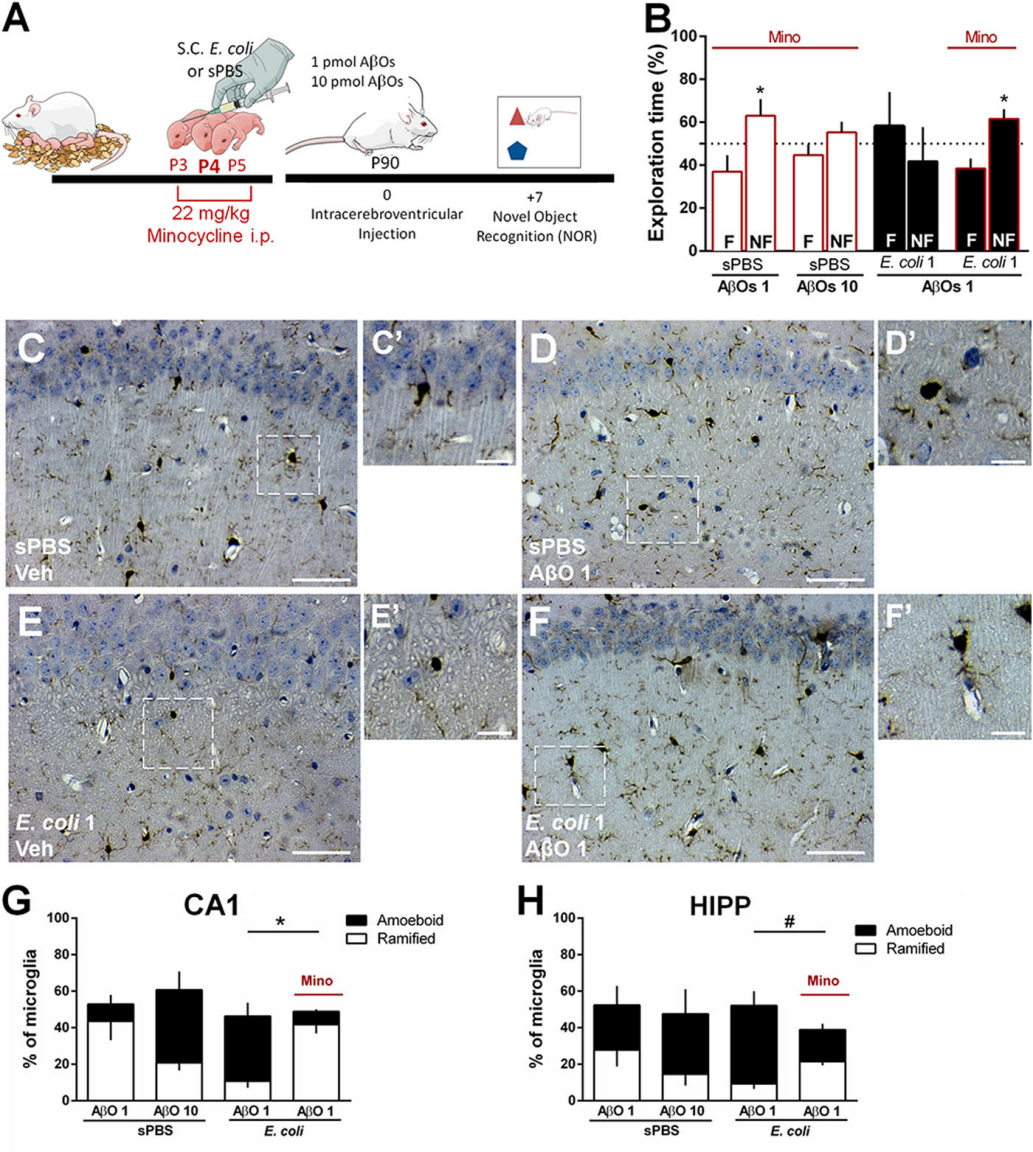
Early blockage of microglial M1 polarization prevents increased susceptibility to AβO-induced microglial activation and cognitive impairment. **a** Neonatal pups received i.p. injection of minocycline (22 mg/kg) between P3 and P5 and, at P4, they also received a s.c. injection of 1 × 10^4^ CFU/g of *E. coli* or sPBS. At P90, mice were treated with an i.c.v. injection of vehicle (Veh), 1 pmol (AβOs 1) or 10 pmol AβOs (AβOs 10). **b** Seven days after AβOs injections animals were tested in NOR task. Bars represent mean ± S.E.M. of percentage of time spent exploring the familiar (F) and non-familiar (NF) objects used in test session. **c**–**f**’ Representative images of Iba-1 immunoreactivity in the DG hippocampal subregion of mice subjected to *E. coli* 1 or sPBS s.c. injection at P4, and given Veh, 1 or 10 pmol of AβOs i.c.v. at P90. Graphs show the percentage of microglial cells that show an amoeboid or ramified morphology in the CA1 (**g**) and total hippocampus (**h**). Scale bar in (**c**, **d**, **e** and **f**): 50 µm; scale bar in (**c**’, **d**’, **e**’ and **f**’): 20 µm. In (**b**) *p = 0.0133 for sPBS + AβOs 1 and *p = 0.009 for *E. coli* 1 + AβOs 1 + Mino, Student’s *t* test compared to fixed value 50. In (**g**): *p = 0.0287 and in (**h**): #*p* = 0.0553

We hypothesized that the transient neonatal brain inflammation after *E. coli* infection in mice is crucial for microglial priming and drive the increased susceptibility to memory impairment following exposure to subtoxic doses of AβOs. To address this hypothesis, polarization of myeloid cells to an M1 profile was blocked with minocycline (22 mg/kg i.p.), administered between P3 and P5 (Fig. 5a). Minocycline had no effect on NOR performance of mice given AβOs 1 or 10 pmol (Fig. 5b, white bars with red borders). Interestingly, neonatal minocycline treatment rescued normal memory performance in mice subjected to *E. coli* infection and exposed to subtoxic doses of AβOs (Fig. 5b, black bars with red borders). Next, we evaluated the morphology of microglial cells in minocycline-treated *E. coli* infected mice in response to AβOs. A significant decrease in the percentage of microglial cells with an amoeboid morphology was found in infected mice treated with minocycline compared to infected mice treated with saline, when exposed to subtoxic doses of AβOs i.c.v. (Fig. 5c–h). This effect was more pronounced in the CA1 (Fig. 5c–g) and CA3 hippocampal regions (Suppl. Fig. 7A–E) and even though in the DG results did not reach statistical significance (Suppl. Fig. 7F–J), a protective effect of minocycline was still observed when data from all hippocampal subregions were averaged (Fig. 5h). Altogether, these results strengthen the hypothesis that microglial priming following *E. coli* infection in neonatal mice might account for the increased susceptibility to AβO-induced microglial activation, synapse loss and cognitive impairment later in life.

## Discussion

Historically, the brain was regarded as an immunologically privileged site^27^. However, increasing evidence suggest that several peripheral pro-inflammatory conditions can be signaled through the blood-brain barrier^28^. Studies demonstrate that even mild systemic pathogenic infections can be deleterious to the brain, especially when occurring during vulnerable periods, such as during development^3,29^ or aging^30,31^. Of interest, experiments in animal models showed that neonatal insults, as ischemia, stroke or severe infections, were associated to altered brain response which persist until adulthood^32^. Here, we developed a model of neonatal s.c. *E. coli* infection that causes no mortality, body weight loss or detectable behavioral alterations in adult mice. Viable *E. coli* particles were found in blood of mice for at least 24 h after s.c. injection, indicating that this model is associated to systemic infection. Bacterial RNA was found in the brains of infected mice at a very early timepoint post-infection (1 h) and no consistent increase in blood brain barrier permeability was detected up to 24 h after infection. Bacteria dynamics in brain samples were reproduced in saline-perfused mice, suggesting that *E. coli* detected by qPCR did not correspond exclusively to circulating bacteria. Therefore, it is possible that *E. coli* reached the brain parenchyma without causing BBB disruption (possibly inside immune cells), or injected bacteria remains adhered to the epithelial lining of the choroid plexus and brain ventricles, since intense binding of *E. coli* was shown especially in neonatal rodents^33^. We further found that *E. coli* infection induced a transient shift to a pro-inflammatory profile in the developing brain. Our results are in agreement with previous studies that reported increased brain levels of pro-inflammatory cytokines within hours^34–40^ after perinatal exposure to gram negative bacteria or LPS. In contrast to the effect of neonatal infections, we found that *E. coli* infection during later stages of development does not lead to hippocampal astrogliosis and microgliosis in mice. Altogether, these results strengthen the idea that there is a critical stage during development when the brain is more sensitive to the effects of pro-inflammatory peripheral stimuli.

The observation that systemic inflammatory diseases can contribute to sporadic AD onset^41^ has led to the hypothesis that periphery-derived pro-inflammatory molecules could also influence brain response to both physiological and pathological conditions^42^. To date, a few clinical studies have suggested the existence of a relationship between systemic infections and different hallmarks of AD. The incidence of two or more unspecific infections over a period of 4 years was associated with an increased risk of AD^43^, and a single episode of pneumonia resulted in an accelerated development of dementia in elderly^44^. Other groups have used transgenic mouse models of AD to investigate whether perinatal infections contribute to AD progression^45^. It is important to note that these studies were performed in animal models mimicking genetic, familial variants of AD, which are not always good representatives of sporadic AD development in humans^46^. Of note, studies in healthy human subjects and mice have shown that Aβ peptide is constantly generated under physiological conditions in the brain^47–49^.Moreover, since AD pathology is known to start many years before the clinical onset of symptoms, it is important to investigate whether systemic infections contribute to increase the risk of AD development, or only accelerate ongoing disease. Here, we report that a single exposure to *E. coli* during the neonatal period is sufficient to increase the susceptibility of mice to cognitive impairment and synapse loss after a single i.c.v. infusion of subtoxic doses of AβOs. Therefore, we speculate that early life infections could increase the risk to develop mild-cognitive impairment and AD, depending on the individual response to physiological generation of Aβ in the patients’ brains. This hypothesis should be further confirmed by additional epidemiological and experimental studies. Moreover, the timing of an immunological challenge is very important when considering possible lasting effects. Our results show that mice infected during adolescence or adulthood showed normal cognitive performance when AβOs are infused into the brain, in contrast to what was found following neonatal infection. Previous studies have found that *E. coli* adheres significantly more to the choroid plexus of neonatal rats compared to that of adult animals, under identical experimental conditions^33^. In agreement with these findings, we found that when adolescent mice are infected s.c. with *E. coli*, bacteria are not detectable in the brain by qPCR either 1 or 24 h after infection, in contrast to our results following infection in neonatal mice. These findings highlight that infections during the neonatal period have larger potential to cause long-lasting impact on brain and behavior.

Despite the evidence of serious late effects caused by early-life infections on the developing brain, the underlying mechanisms are not well understood. Primed microglial cells are more susceptible to adopt a pro-inflammatory state when exposed to a second mild inflammatory hit, a phenomenon that was shown to occur in vivo following early-life exposure to LPS or *E. coli* in rodents^25^. Recent evidence support a central role of microglial cells in performing phagocytosis of dysfunctional synapses^50^, in several conditions including AD^51^. Here, we showed that microglial cells from *E. coli*-infected pups become over responsive and undergo exaggerated activation when exposed to subtoxic doses of AβOs, in vitro and in vivo, leading to synapse loss and cognitive impairment. Similar results were seen in all hippocampal subregions, suggesting that there is not a subpopulation of microglial cells which is particularly sensitive to the effects of neonatal *E. coli* infection. In addition, we found that by blocking microglial activation shortly after infection with minocycline, we were able to restore normal susceptibility of these cells when animals were exposed to subtoxic doses of AβOs later in life, and this also led to normalized memory function in the presence of low doses of Aβ oligomers. These results strengthen the hypothesis that neonatal infection induces microglial priming, and that overactivation of these primed cells in response to low amounts of AβOs later in life leads to cognitive impairment.

It is known that early-life stressors may have differential impacts on subjects of both sexes^52,53^. One limitation of our study is that we used only male mice in behavioral evaluations. This was due to fact that, under our conditions, female pups were more susceptible to long-term cognitive effects of early-life *E. coli* infection, and further studies should focus on the sex-specific effects of *E. coli* on the developing brain.

Altogether, our findings highlight the need for special attention to the potential late burden of early life bacterial infections. Our data provide mechanistic insight into the long-term consequences of neonatal infections and suggest that these conditions might be associated to increased susceptibility to neurodegenerative diseases later in life. Further efforts should be targeted to the discovery of pharmacological approaches that could hamper in vivo infection-induced microglial priming and thus prevent its late deleterious effects to the brain.

## Materials & methods

### Experimental design

The aim of this study was to evaluate whether early-life infection can increase susceptibility to cognitive impairment induced by AβOs. All pups in each litter received the same treatment to avoid cross-contamination, and litters were assigned to a single group by simple randomization. Researchers were blind to experimental conditions when conducting the experiments and analyzing the results.

### Statistical analyses

Statistical analysis was performed using GraphPad Prism 6.01 (GraphPad, San Diego, CA). Data are reported as means ± S.E.M. Gaussian distribution of data was assessed using the D’Agostino-Pearson normality test. For data with Gaussian distribution, the Rout’s test was employed to identify potential outliers, which were then excluded from further analyses.

Data from the novel object recognition test were analyzed by one-sample *t* test compared to the fixed value of 50. For all other data, either unpaired Student’s t test or one-way ANOVA followed by Tukey’s post hoc test was used. p values are indicated in the corresponding figure legend.

### Animals and mating

Naïve 10 weeks-old Swiss mice were obtained from our breeding colony and mated for 2 weeks (four females per male). After this period, males were removed and females were kept in individual cages until the birth of the offspring. All procedures followed the *Principles of Laboratory Animal Care* from the National Institutes of Health and were approved by the Institutional Animal Care and Use Committee of the Federal University of Rio de Janeiro (protocol #049/14).

### Subcutaneous Escherichia coli infection

Male and female pups received s.c. injection of sterile PBS (sPBS) or different doses of *E. coli* ATCC 25922 in a total volume of 0.05 mL at post-natal days 4 (P4), 21 (P21) or 60 (P60). For *E. coli* injection, all pups were removed from the mother at the same time, placed into a clean cage with bedding, injected individually and returned to the mother as a group. The whole procedure did not take longer than 5 min. All littermates received the same treatment in order to avoid cross-contamination. *E. coli* injection triggered no mortality, independent of concentration. In an independent experiment, maternal care was evaluated in *E. coli* and sPBS groups from post-natal day 2 to post-natal day 8, according to the procedure described by Reis and colleagues (2014). At P21, mice were weaned and placed with same-sex littermates (2–5 mice per cage). Mice were housed in polypropylene cages and maintained at 25 °C with controlled humidity, under a 12 h light/dark cycle and ad libitum access to water and chow. To control for litter effects, a maximum of two pups per litter across a minimum of 4 different litters were assigned to a single experimental group.

### Preparation of AβOs

Aβ oligomers were prepared weekly from synthetic Aβ_1–42_ (American Peptide, Sunnyvale, CA) as described previously^19,54^. The peptide was dissolved in 1 mM hexafluoroisopropanol (HFIP), which was then evaporated for the formation of a dried film that was stored at −80 °C. Films were then dissolved in DMSO to a final concentration of 5 mM, followed by ice-cold PBS and incubated overnight at 4 °C. After incubation, the preparation was centrifuged (14,000 × *g*, 10 min) and supernatant was collected. Preparations were routinely characterized by HPLC size-exclusion chromatography (SEC) under non-denaturating conditions. Occasionally, preparations were also characterized by western immunoblots using anti-Aβ, 6E10 (Abcam, Cambridge, MA, cat#ab54518) or anti-Aβ oligomer NU4^55^, as previously described^56–58^. SEC analysis reveals that AβO preparations comprise a mixture of high-molecular weight (molecular masses ranging from 80 to 150 kDa) and low-molecular weight (average molecular mass ~ 10 kDa) oligomers^19^. Protein concentration was determined using the BCA kit (Thermo Fisher Scientific, Waltham, MA). Oligomers were kept at 4 °C and used within 48 h of preparation.

### Evaluation of maternal behavior

Maternal behavior was evaluated between post-natal days 2 and 8, according to the procedure described by Reis and colleagues (2014). Briefly, the percentage of time spent by dams licking or feeding pups, and percentage of time they spent at the nest were assessed in four 1 h-long observation sessions performed each day at the following hours of the day: 8:00 AM, 1:00 PM, 4:00 PM and 7:00 PM.

### Intracerebroventricular (i.c.v.) injections

For i.c.v. injections, animals were anesthetized for 7 min with 2.5% isoflurane (Cristália, Itapira, Brazil) using a vaporizer system and gently restrained only during the injection procedure itself, as described^19,59^. A 30 G 2.5 mm-long needle was unilaterally inserted 1 mm to the right of the midline point equidistant from each eye and 1 mm posterior to a line drawn from the anterior base of the eye. AβOs (1 or 10 pmol) or vehicle were injected in a final volume of 3 µl, and the needle was kept in place for 30 s to avoid backflow. Accurate placement of the needle into the right lateral ventricle was confirmed by macroscopic examination of dissected brains before immunohistochemistry. Mice showing any signs of misplaced injections or brain hemorrhage (~ 5% of animals throughout our study) were excluded from further analysis.

### Minocycline treatment

When indicated, neonatal mice received daily i.p. injections of minocycline (Sigma-Aldrich, St. Louis, MO, cat# M9511; 22 mg/kg)^60,61^ or saline between post-natal days 3 and 5.

### Novel object recognition test

The novel object recognition test was performed in an open field arena measuring 30 × 30 × 45 cm, where objects were fixed to the box using tape, as described^19^. Preliminary tests showed that none of the objects used in our experiments evoked innate preference. Before training, each animal was submitted to a 5-min-long habituation session, in which they were allowed to freely explore the empty arena. During this habituation session, total distance traveled was measured using Anymaze software (Stoelting Co, Wood Dale, IL), to verify possible effects of treatments on locomotor behavior. Training consisted of a 5 min-long session during which animals were placed at the center of the arena in the presence of two identical objects. Time spent exploring each object was recorded. Sniffing and touching the objects were considered as exploratory behavior. The arena and objects were thoroughly cleaned between trials with 70% ethanol to eliminate olfactory cues. Two hours after training, animals were again placed in the arena for a 5 min-long test session, and one of the objects used in the training session was replaced by a new one. Again, time spent exploring each object was recorded. Results were expressed as percentage of time exploring each object during the training or test sessions and were analyzed using a one-sample Student’s *t* test comparing the mean exploration time for each object with the fixed value of 50 (50%, i.e., no differentiation between objects). By definition, animals that recognize the familiar object as such (i.e., learn the task) explore the novel object > 50% of the total time.

### Immunohistochemistry

Mice were deeply anaesthetized with 1.5 mL/kg of a solution containing 10% ketamine and 2% xylazine (intraperitoneally). They were then transcardially perfused with cold phosphate-buffered saline (PBS) solution followed by fresh 4% formaldehyde. Brains were removed, post-fixed for 24 h in 4% formaldehyde and embedded in paraffin after dehydration and diaphanization. Paraffin-embedded brain tissue sections (3–5 µm) were immersed in xylene for 10 min, rehydrated in absolute ethanol followed by 95 and 70% solutions of ethanol in water. Another set of samples were cryopreserved after perfusion, and slides with frozen coronal brain sections (40 μm-thickness) were fixed in acetone for 30 min. In order to inactivate endogenous peroxidase, paraffin-embedded or frozen slices were incubated with 3% H_2_O_2_ in methanol. Antigens were reactivated by treatment with 0.01 M citrate buffer for 40 min at 95 °C. Slides were washed in PBS and incubated with primary antibodies (rabbit anti-GFAP antibody, 1:500, Agilent cat# Z0334, RRID:AB_10013382; rabbit anti-Iba-1 antibody, 1:1000, Wako cat# 016–20001, RRID:AB_839506; rabbit anti-Ki-67, 1:50, Abcam cat# ab15580, RRID:AB_443209; mouse anti-synaptophysin, 1:500, Sigma-Aldrich cat# S5768, RRID:AB_477523) for 12–16 h at 2–8 °C. After washing with PBS, slides were incubated with biotinylated secondary antibodies for 1 h at room temperature, washed twice with PBS and incubated with streptavidin-biotin-peroxidase (Vector Laboratories, Burlingame, CA) for 30 min. Slides were then covered with 3,3′-diaminobenzidine solution (0.06% DAB in PBS containing 2% DMSO and 0.018% H_2_O_2_) for 1 to 5 min or until a brown precipitate could be observed. Identical conditions and reaction times were used for slides from different animals (run in parallel) to allow comparison between immunoreactivity optical densities. Reactions were stopped by immersion of slides in distilled water. Counter-staining was performed with Harris hematoxilin. Slides were imaged using a Sight DS-5M-L1 digital camera (Nikon, Melville, NY) connected to an Eclipse 50i light microscope (Nikon) at x200 magnification.

One image from each hippocampal subfield (CA1, hilus, DG or CA3) and in the subventricular zone (SVZ) in each hemisphere were obtained using 200x magnification, and an optical density threshold that best discriminated staining from background was defined using ImageJ (NIH, Bethesda, MD; RRID:SCR_003070), as described^62^. Total pixel intensity was determined for each image and data are expressed as integrated optical density (OD).

For determination of microglial morphology, immunostaining for Iba-1 was performed as described above, in brains of mice 24 h after i.c.v. injection of vehicle or AβOs (1 or 10 pmol). Each subject was evaluated in duplicate and the total number of microglial cells was quantified in the hippocampus (CA1, hilus, DG and CA3), using 2 images per hippocampal subregion. Cells were then further classified as ramified (>3 primary ramifications), amoeboid (no primary processes) or intermediary (1–3 ramifications).

For endogenous IgG staining, mice were deeply anesthetized and transcardially perfused as described above. Brains were rapidly removed from skulls, postfixed in PFA 4% for 24 h and cryoprotected in sucrose 20% (w/v). Brains were mounted in OCT, frozen at −20 °C and 20 µm-thick sections were obtained in a cryostate (Leica). Slides containing two to four sections were washed with 0.3% Triton X-100 in PBS (3 × 5 min), followed by an overnight incubation with a biotinylated goat anti-mouse IgG (H + L) antibody (1:200, Vector Laboratories, cat# BA-9200, RRID:AB_2336171). Binding was visualized using the peroxidase-based Vectastain ABC kit (Vector Laboratories, cat# PK-6100, RRID:AB_2336819) and 3,3′-diaminobenzidine (Vector Laboratories, cat# SK-4100, RRID:AB_2336382). Tissues were thereafter washed with PBS (3 × 5 min), dehydrated through graded concentrations of alcohol, cleared in xylene and mounted on Entellan (Merck Millipore, cat# 1079610100). As a positive control of reaction conditions, sections from adult AG129 mice infected with the Zika virus African MR766 strain^24^ were run in parallel.

### Bacteremia assessment

For bacteremia assessment, blood was collected under sterile conditions from anesthetized mice at different times post-infection. Heparin was used to avoid clotting. Blood samples were plated on dishes covered with non-selective Brain Heart Infusion (BHI) + 5% Agar and incubated at 37 °C for 24 h. After incubation, number of colony-forming units (CFU) were counted for estimation of the number of CFU per milliliter of blood. Each sample was plated in duplicate.

### Microglial cultures

For microglial cultures, half of the pups in each litter received a s.c. injection of *E. coli* (1 × 10^4^ CFU/g) ant the other half received an equal volume of sPBS. Twenty four hours later, pups were killed and their brains were collected. Meningeal layers were carefully removed and the hippocampus and cortices were dissected, homogenized in Dulbecco’s Modified Eagle’s Medium (DMEM)-F12 medium (Invitrogen, Carlsbad, CA) and centrifuged (1500 rpm, 2 min, 4 °C). Pellets were solubilized in DMEM-F12 medium enriched with 10% fetal bovine serum, penicillin, streptomycin, and fungizone (0.65 μM; all from Sigma Aldrich, St. Louis, MO). Cells were cultured in 75 cm^2^ cell culture bottles (Corning Glass, Corning, NY) which were pretreated with poly-L lysine overnight (Sigma Aldrich). Cells were maintained at 37 °C in a humidified chamber with 5% CO_2_ for 14 days. Then, the microglial cells were isolated by vigorous shaking for 45–60 min as previously described^40^, resulting in a purified culture consisting of 95% of microglial cells. Microglia were then transferred to 24-well plates (1 × 10^5^ cells/well) with glass coverslips pretreated with poly-L lysine, and cultured with DMEM-F12 medium supplemented with 10% fetal bovine serum. Twenty-four hours later, cells were treated with subtoxic amounts of AβOs (30 nM), determined based on previous work from our group^63^, or an equivalent volume of vehicle.

### Immunocytochemistry

For immunocytochemistry, culture medium was removed and cells were fixed (paraformaldehyde 4% for 15 min), blocked (PBS, Triton 0.2%, 10% bovine serum for 1 h) and incubated overnight with anti-F4/80 (1:200, Abcam cat# ab6640, RRID:AB_1140040) at 4 °C. Secondary antibody (anti-rabbit Alexa 488, 1:1 000, Thermo Fisher Scientific cat# A32723, RRID:AB_263375) was incubated for 2 h, at room temperature. Nuclei were counterstained with DAPI (Sigma Aldrich, cat# D9542). Coverslips were imaged on a Zeiss Axio Observer Z1 microscope.

### qPCR

Total RNA was isolated from whole brains of mice with or without transcardiac perfusion with 5 mL of sterile saline (indicated throughout Results section and Figure Legends), using TRIzol Reagent (Invitrogen, cat# 15596026) according to manufacturer’s instruction. Purity and integrity of RNA were determined by the 260/280 nm absorbance ratio and by electrophoresis in agarose gels. Only preparations with ratios >1.8 and no sign of RNA degradation were used. One µg of RNA was treated with Ambion DNase I RNase-free (Invitrogen, cat# AM2222) before cDNA synthesis (High Capacity cDNA reverse transcription kit, Invitrogen, cat# 4368814) according to manufacturer’s instructions. Expression of genes of interest was analyzed by qPCR on an Applied Biosystems 7500 RT-PCR system using the Power SYBR kit (Applied Biosystems, cat# A25742). Primer pairs were: Act: Fw TGTGACGTTGACATCCGTAAA and Rv GTACTTGCGCTCAGGAGGAG, rRNA 16 s: Fwd TCGTCAGCTCGTGTYGTGA and Rv CGTAAGGGCCATGATG. Cycle threshold (Ct) values were used to calculate fold changes in gene expression using the 2^–ΔCt^ method. In all cases, reactions were performed in 15 µl volume.

### Cytokine quantification

For cytokine quantification, mice were killed by decapitation and brains were rapidly removed, frozen in liquid nitrogen and kept at −80 °C until all samples had been collected. Brains without the cerebellum were homogenized in PBS with protease and phosphatase inhibitors (Thermo Fisher Scientific, cat# 78440) on ice and centrifuged at 15,000 rpm, at 4 °C for 10 min. Supernatants were collected and run in duplicate for IL-1β (Thermo Fisher Scientific cat# 88–5019–22, RRID:AB_2574805) and TNF-α (Biolegend, San Diego, CA, cat# 430902) following the manufacturer’s instructions. Total protein in each sample was determined with the Pierce BCA protein assay kit and used for normalization.

## Supplementary information

Supplementary Information
