# Supplementary material for: Neonatal infection leads to increased susceptibility to Aβ oligomer-induced brain inflammation, synapse loss and cognitive impairment in mice
Source: Cell Death Dis. 2019 Apr 11;10(4):323. doi: 10.1038/s41419-019-1529-x (PMC6459845; doi:10.1038/s41419-019-1529-x)
Supplement: Supplementary file 1 — Supplementary Information [file 41419_2019_1529_MOESM1_ESM.docx]

**Frost et al., Supplementary Information**

**
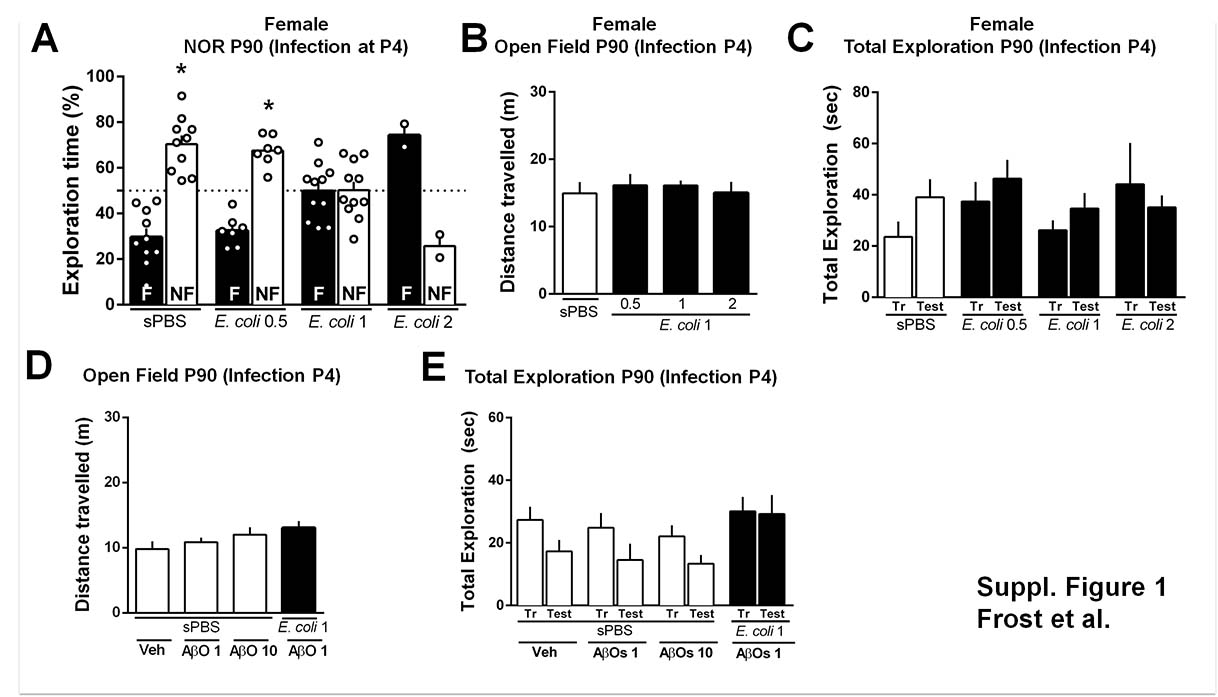
**This file contains seven supplementary figures and their corresponding legends.

**Supplementary Figure 1. Female pups are more susceptible to persistent cognitive impairment following neonatal *E. coli* infection.** (**A**) Swiss female pups received a subcutaneous (s.c.) injection of sterile PBS (sPBS) or 0.5, 1 or 2 x10^4^ CFU/g of body weight of *Escherichia coli* (*E. coli* 0.5, *E. coli* 1, *E. coli* 2, respectively) at post-natal day 4 (P4). At post-natal day 90 (P90), they were tested in the Novel Object Recognition (NOR) task. Bars represent mean ± S.E.M. of percentage of time spent exploring de familiar (F; black bars) and non-familiar (NF; white bars) objects used in test session. (**B**) Distance travelled by female mice in the open field arena at P90. (**C**) Total exploration by female mice during training and test sessions. (**D**) Distance travelled by male mice in the open field arena at P90. (**E**) Total exploration by male mice during training and test sessions. Bars represent mean ± S.E.M. In A: *p=0.0005 for sPBS and 0.0004 for *E. coli* 0.5. Student’s *t* test compared to fixed value 50.

**
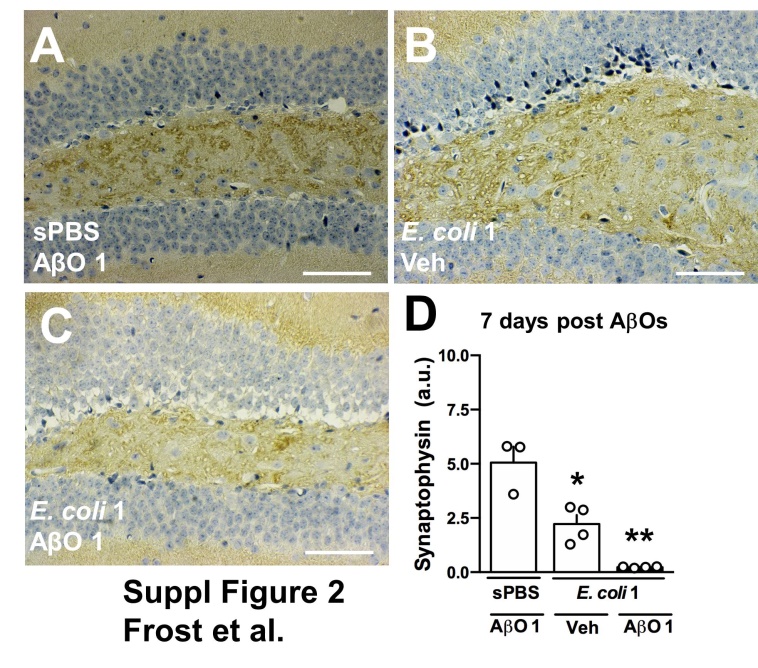
**

**Supplementary Figure 2. Neonatal *E. coli* infection increases susceptibility to AβO-induced synapse loss.** (**A-C**) Representative images of synaptophysin immunoreactivity in the DG hippocampal subregion of mice subjected to *E. coli* 1 infection or sPBS at P4, and given an i.c.v. injection of vehicle or 1 pmol AβOs (AβO 1). Brains were analyzed 7 days after i.c.v. injection of AβOs. (**D**) Graph shows integrated immunoreactivity (optical density) for synaptophysin in the hippocampus 7 days after AβOs injection. Scale bar: 50 µm. *p=0.0047, **p<0.0001, one-way ANOVA followed by Tukey.


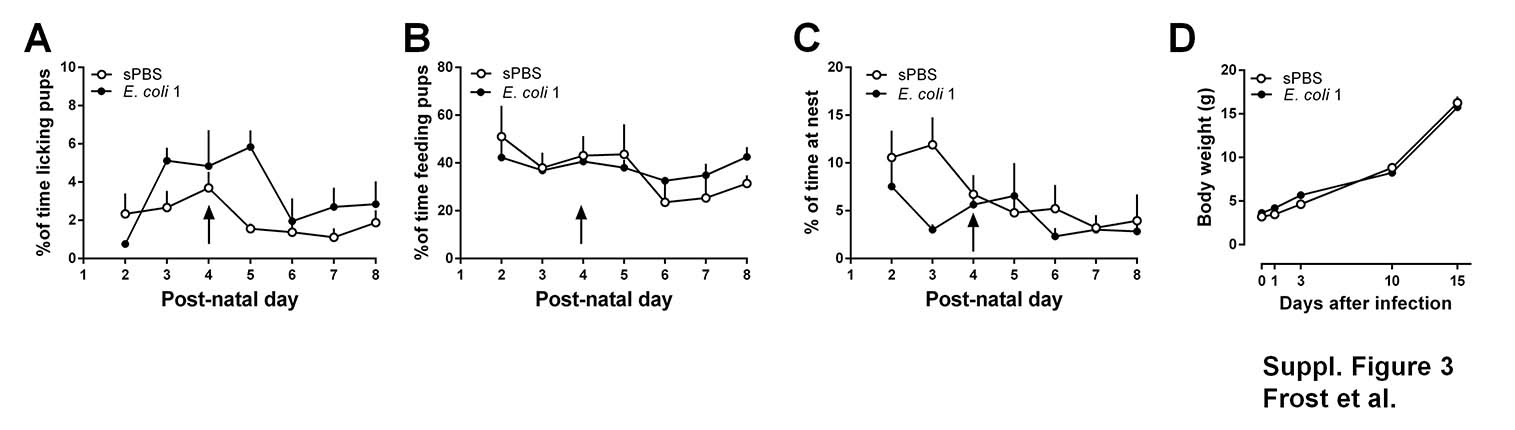
**Supplementary Figure 3. Neonatal *E. coli* infection does not affect maternal behavior or body weight gain of pups.** Swiss mice were subjected to a s.c. injection of 1x10^4^ CFU/g of *E. coli* (*E. coli* 1) or sterile PBS (sPBS) at post-natal day 4. (**A-C**) Maternal behavior was evaluated as described in Methods, for two days before and four days after infection for the following parameters: percentage of time that dams spent licking pups (**A**), percentage of time spent feeding pups (**B**) and percentage of time at the nest (**C**). (**D**) Body weight of pups was measured at different times after s.c. injection of sPBS or *E. coli*. Arrows indicate the day of *E. coli* or sPBS s.c. injection. N = 4 dams/group.

**
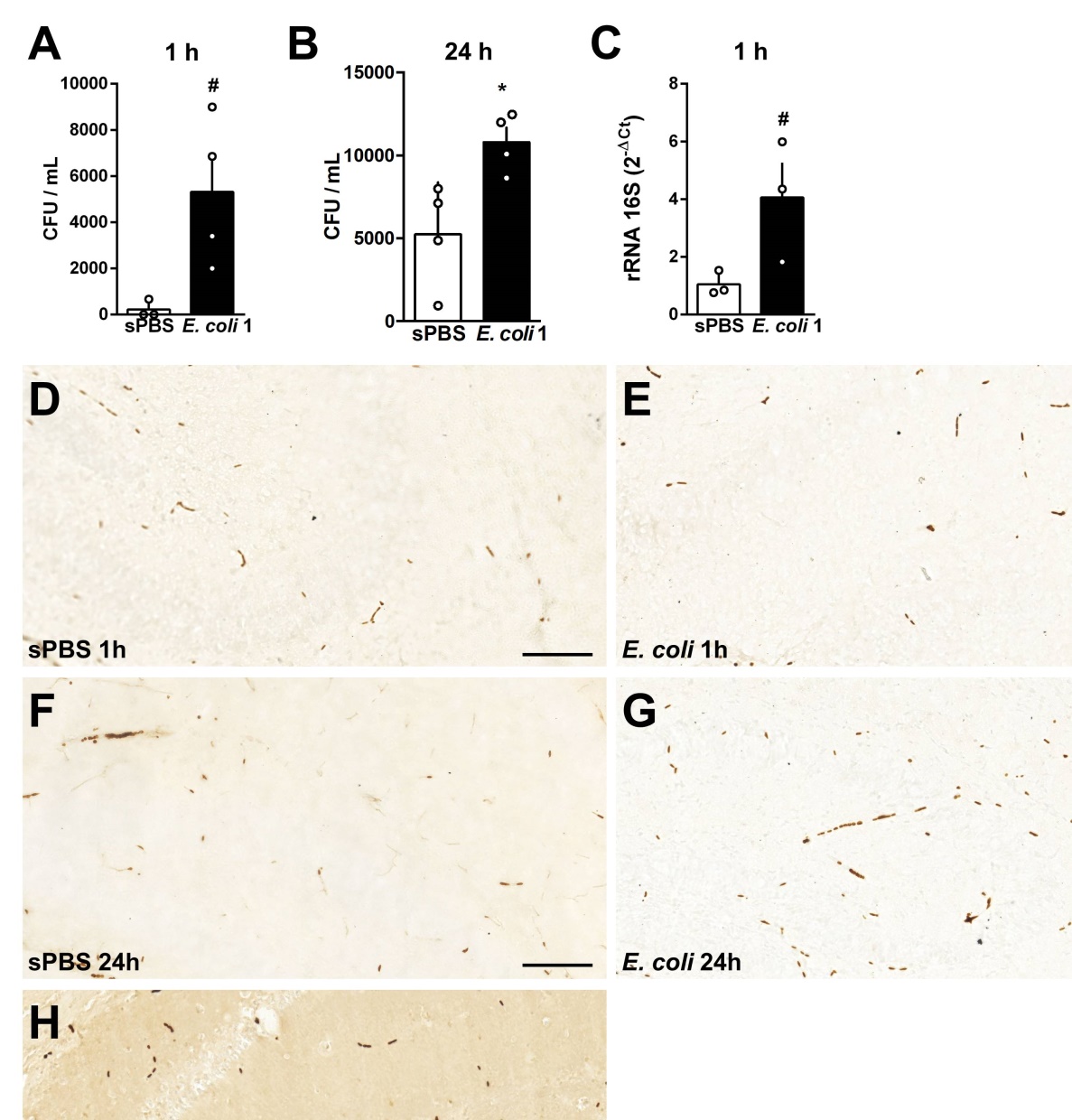
**

**
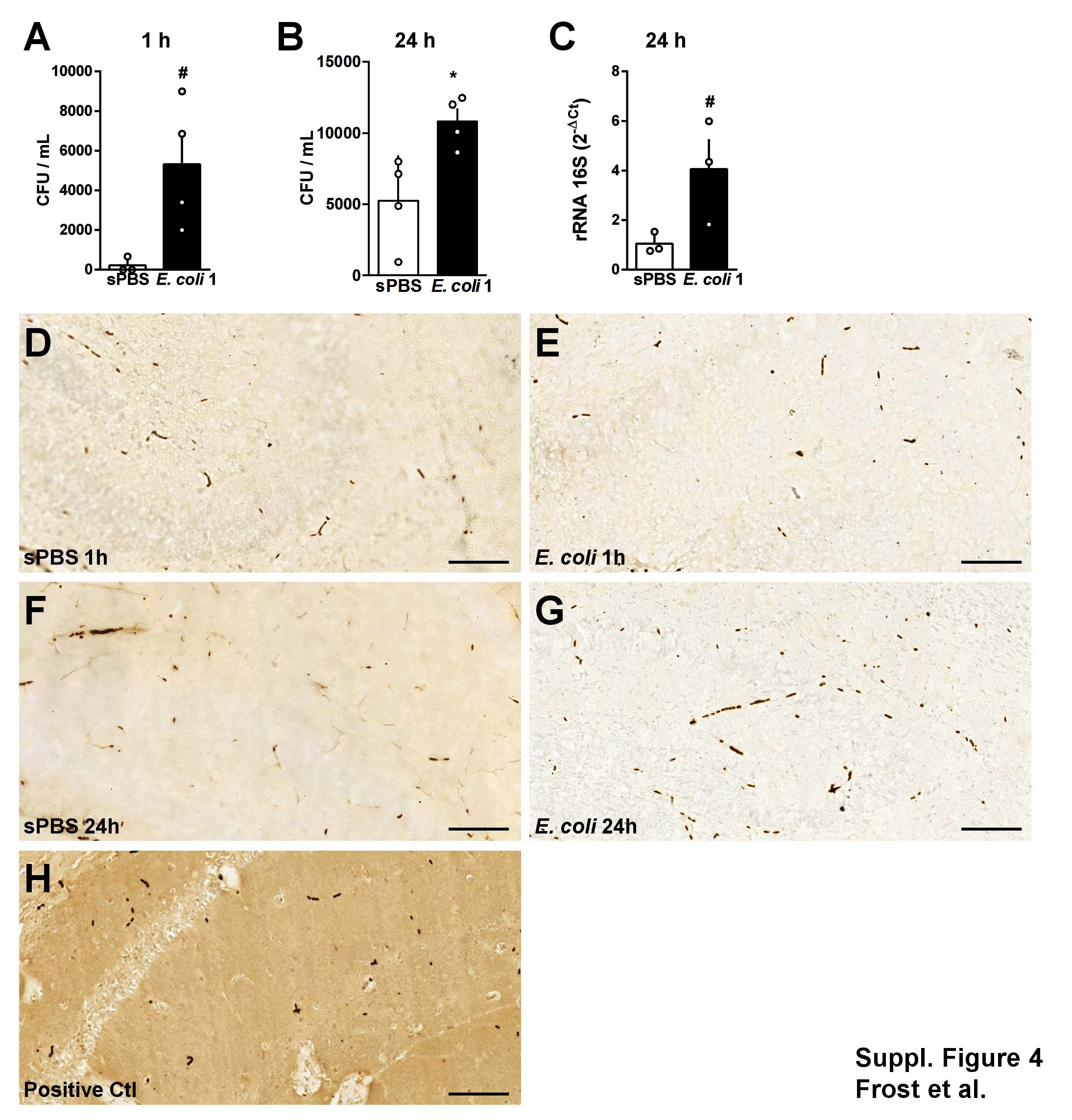
**

**Supplementary Figure 4. Neonatal *E. coli* infection does not disrupt the blood brain barrier (BBB).** Swiss pups received a s.c. injection of sterile PBS (sPBS) or *E. coli* 1x10^4^ CFU/g (E. coli 1) at post-natal day 4 (P4) and bacteremia was determined 1h (**A**) or 24h (**B**) later. (**C**) qPCR for gamma-proteobacteria rRNA 16S was performed in saline-perfused brains of mice 1 h after s.c. injection of *E. coli* or sPBS, and normalized by actin. (**D-H**) Representative images of immunohistochemistry performed using anti-mouse IgG antibody to evaluate BBB integrity. (**H**) Brain sections from an adult AG129 mouse infected with the African MR766 ZIKV strain were used as positive control. Scale bar: 100 µm.

**
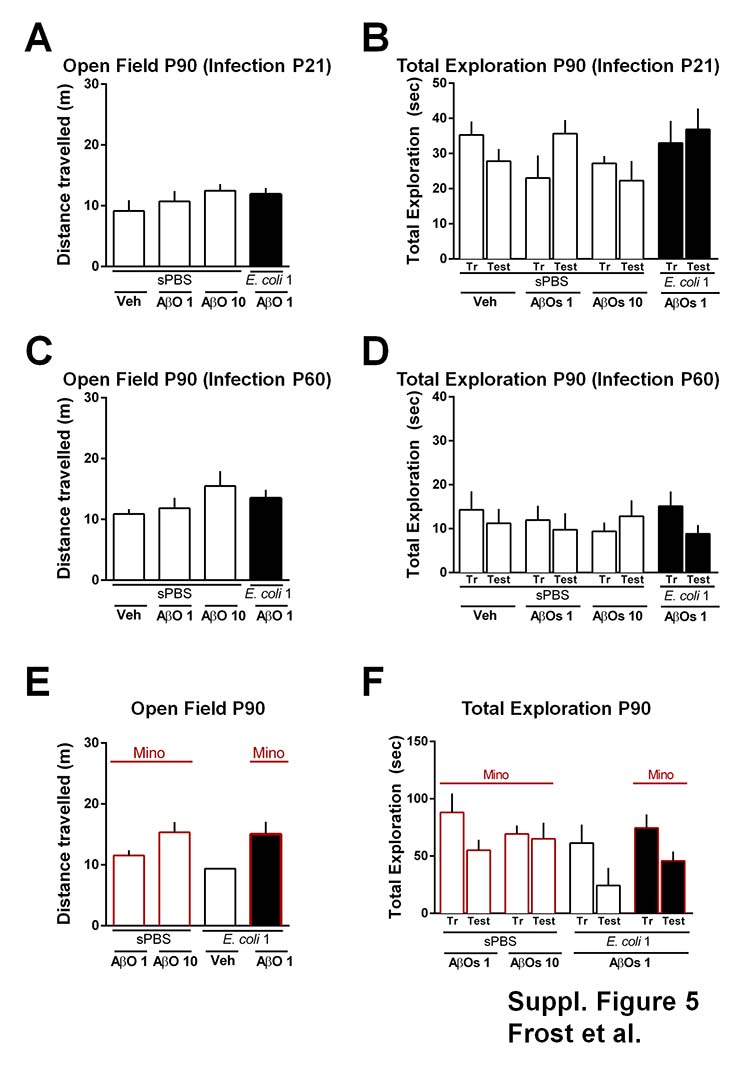
**

**Supplementary Figure 5. Open field and total exploration for adult mice.** (**A**, **C, E**) Distance travelled in the open field at P90 of animals injected s.c. with 1x10^4^ CFU/g of *E. coli* (*E. coli* 1) or sterile PBS (sPBS) at P21 (**A**), P60 (**C**) or P4 (**E**). (**B**, **D, F**) Total exploration during NOR training and test sessions at P90 of mice injected s.c. with 1x10^4^ CFU/g of *E. coli* (*E. coli* 1) or sterile PBS (sPBS) at P21 (**B**), P60 (**D**) or P4 (**F**). For data shown in **E** and **F**, mice were treated with minocycline i.p. (22 mg/kg) between P3 and P5 (see Methods). Bars represent mean ± S.E.M.


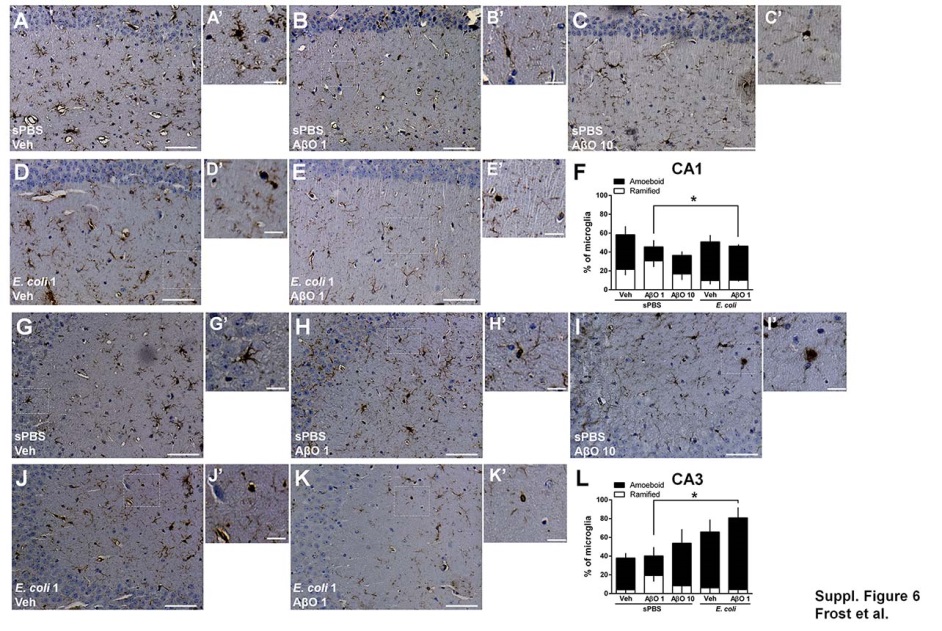


**Supplementary Figure 6. Microglial cells from neonatal *E. coli*-infected mice show increased susceptibility to AβOs.** Swiss pups received a s.c. injection of sterile PBS (sPBS) or *E. coli* 1x10^4^ CFU/g (*E. coli* 1) at post-natal day 4 (P4). At P90, animals received an i.c.v. injection of vehicle (Veh), 1 pmol (AβOs 1) or 10 pmol AβOs (AβOs 10) at P90. (**A**-**E’**) Representative images of Iba-1 immunoreactivity in the CA1 hippocampal subregion of mice subjected to *E. coli* 1 or sPBS s.c. injection at P4, and given vehicle, 1 or 10 pmol of AβOs i.c.v. at P90. (**F**) Graph shows the percentage of microglial cells that show an amoeboid or ramified morphology in the CA1 hippocampal region. (**G**-**K’**) Representative images of Iba-1 immunoreactivity in the CA3 hippocampal subregion of mice subjected to *E. coli* 1 or sPBS s.c. injection at P4, and given Veh, 1 or 10 pmol of AβOs i.c.v. at P90. (**L**) Graph shows the percentage of microglial cells that show an amoeboid or ramified morphology in the CA3 hippocampal region. Scale bar: 50 µm; scale bar insets: 20 µm. In (**F**) *p=0.0196; in (**L**) *p=0.0086, Student’s t test.


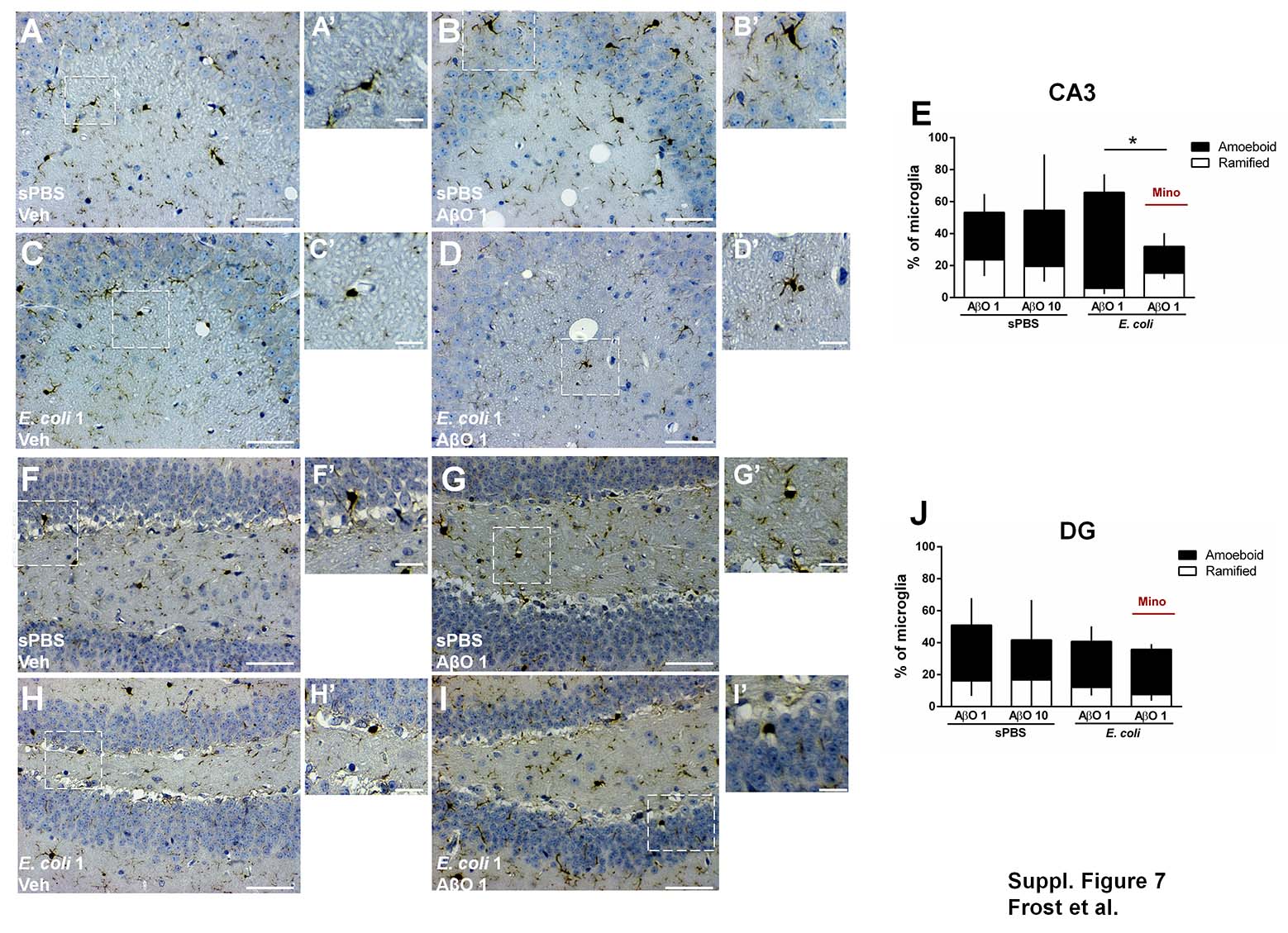
**Supplementary Figure 7. Early blockage of microglial M1 polarization prevents increased susceptibility to AβO-induced microglial activation in adulthood.** Neonatal pups received i.p. injection of minocycline (22 mg/kg) between P3 and P5 and at P4, they also received a s.c. injection of 1x10^4^ CFU/g of *E. coli* or sPBS. At P90, mice were treated with an i.c.v. injection of vehicle (Veh), 1 pmol (AβOs 1) or 10 pmol AβOs (AβOs 10). (**A**-**D’**) Representative images of Iba-1 immunoreactivity in the CA3 hippocampal subregion of mice subjected to *E. coli* 1 or sPBS s.c. injection at P4, and given Veh, 1 or 10 pmol of AβOs i.c.v. at P90. (**E**) Graph shows the percentage of microglial cells that show an amoeboid or ramified morphology in the CA3 hippocampal region. (**F**-**I’**) Representative images of Iba-1 immunoreactivity in the DG hippocampal subregion of mice subjected to *E. coli* 1 or sPBS s.c. injection at P4, and given Veh, 1 or 10 pmol of AβOs i.c.v. at P90. (**J**) Graph shows the percentage of microglial cells that show an amoeboid or ramified morphology in the DG hippocampal region. Scale bar: 50 µm; scale bar insets: 20 µm. In (**E**) *p=0.0376, Student’s t test.
